# Lightweight and High-Stiffness Metal Optical Systems Based on Additive Manufacturing

**DOI:** 10.3390/mi15010128

**Published:** 2024-01-12

**Authors:** Qiang Fu, Lei Yan, Shuanglong Tan, Yang Liu, Lingjie Wang

**Affiliations:** 1 Changchun Institute of Optics, Fine Mechanics and Physics, Chinese Academy of Sciences, Changchun 130033, China; yanlei@ciomp.ac.cn (L.Y.); 15567408408@126.com (S.T.); liu9527aaa@163.com (Y.L.); wanglingjie@126.com (L.W.); 2 University of Chinese Academy of Sciences, Beijing 101408, China

**Keywords:** optical system, metal mirrors, additive manufacturing, local cooling optical system, topology optimization

## Abstract

To build a long-wave infrared catadioptric optical system for deep space low-temperature target detection with a lightweight and wide field of view, this work conducted a study that encompasses a local cooling optical system, topology optimization-based metal mirror design, and additive manufacturing. First, a compact catadioptric optical system with local cooling was designed. This system features a 55 mm aperture, a 110 mm focal length, and a 4-degree by 4-degree field of view. Secondly, we applied the principles of topology optimization to design the primary mirror assembly, the secondary mirror assembly, and the connecting baffle. The third and fourth modes achieved a resonance frequency of 1213.7 Hz. Then, we manufactured the mirror assemblies using additive manufacturing and single-point diamond turning, followed by the centering assembly method to complete the optical assembly. Lastly, we conducted performance testing on the system, with the test results revealing that the modulation transfer function (MTF) curves of the optical system reached the diffraction limit across the entire field of view. Remarkably, the system’s weight was reduced to a mere 96.04 g. The use of additive manufacturing proves to be an effective means of enhancing optical system performance.

## 1. Introduction

Target detection in deep space under low-temperature conditions encompasses a broad field, including activities such as deep space detection and space-based early warning, among other related tasks [1]. In this field, catadioptric optical systems play a significant role [2]. With the continuous development of science and technology, catadioptric optical systems are evolving towards becoming more dexterous, lightweight, and low radiation, with a large field of view and higher resolution capabilities.

In the catadioptric optical system, the reflective optical elements at the front end have witnessed an increasing adoption of metal mirrors, driven by the development of single-point diamond turning (SPDT) technology. This transition is attributed to the benefits offered by their advantages of good processing workmanship and lower material prices, etc. Typical metal mirror materials include aluminum alloys, magnesium–aluminum alloys, beryllium–aluminum alloys, etc. [3,4,5]. With the escalating requirements for lightweight and high stiffness in optical systems, traditional processing methods for aluminum alloy mirrors are increasingly unable to meet these demands, which, to a certain extent, constrain the application of aluminum alloy mirrors. To address these challenges, the technology of aluminum alloy mirrors based on additive manufacturing has emerged and become a hot research topic in recent years [6,7]. Metal mirrors produced through additive manufacturing employ similar processing techniques to traditional metal mirrors. Additionally, the topology optimization methods can be applied to the design of the metal mirror. This innovative approach enables the creation of enclosed metal mirrors that achieve the elusive combination of lightweight and high stiffness, which is often unattainable through traditional means.

In 2015, the Corning Corporation of the United States prepared a honeycomb light and high-performance aluminum mirror through additive manufacturing technology, which improved the processing and forming efficiency compared to the traditional metal mirror matrix preparation [8,9]. The Fraunhofer Institute in Germany proposed a sandwich-closed honeycomb structure, which connects all honeycomb spaces through holes in the internal reinforcing ribs, and the stiffness of the mirror is higher than that of the traditional metal mirror [10]. In 2017, E. Hilpert et al. compared five structural forms of metal mirrors and analyzed the advantages of metal mirrors prepared by additive manufacturing technology [11]. In 2019, E. Hilpert and colleagues further refined the lightweight design of the mirror, achieving a remarkable weight reduction of 60.5% while simultaneously maintaining the required stiffness of the metal mirror [12].

In this paper, we present the development of an optical system tailored to the specific requirements of low-temperature target detection in deep space. We employed the topology optimization method to enhance the design of the reflection system and then utilized additive manufacturing to produce the primary mirror assembly, secondary mirror assembly, and connecting baffle. SPDT was employed for optical processing. To address surface defects on the printed components, we implemented a nickel–phosphorus modification process. As a result, the finalized primary and secondary mirrors exhibit a high surface shape accuracy. Subsequently, the performance of the system was tested after optical mechanical assembly. The test results demonstrate that the MTF of the optical system reaches the diffraction limit across the entire field of view.

## 2. Topology Optimization Design of the Metal Mirror

The topology optimization method is fundamental for a specific design interval, aiming to seek the best distribution for achieving the optimal design configuration. Currently, the widely employed topological expressions include the variable thickness method, homogenization method, and relative density method [13].

The topology optimization design is carried out using the relative density method, which is an enhanced approach based on the homogenization method and takes the relative density of a unit as the design variable. The relative density is set between 0 and 1. A relative density value closer to 1 signifies the higher importance of the unit in the design, implying that the unit should be retained or emphasized. Similarly, when the relative density approaches 0, it suggests that the unit material can be removed. At present, the most frequently used method in the relative density method is the isotropic material with penalization (SIMP), which is widely used in many general finite element analysis software. The relation between the elastic modulus of unit and the relative density is given by

(1) $$E_{i}=\left[\alpha_{0}+\left(\alpha_{1}-\alpha_{0}\right){\alpha_{i}}^{P}\right]E_{0},i=1,2,\cdots ,N$$

where $E_{i}$ is the elastic modulus, $\alpha_{i}$ is the relative density of unit, $\alpha_{0}$ is the lower limit of the relative density of unit, $\alpha_{1}$ is the upper limit of the relative density of unit, $E_{0}$ is the elastic modulus of materials, N is the number of unit in the design interval, and P is the penalty factor.

Here, by setting the topology optimization unit threshold $\alpha_{C}$ and comparing the size of $\alpha_{i}$ and $\alpha_{C}$ , the units in the design interval are selected. Here, the design variables can be expressed as

(2) $$\alpha ={\left(\alpha_{1},\alpha_{2},\cdots ,\alpha_{N}\right)}^{T}$$

Based on the above, the topology optimization design of the mirror support structure is undertaken, and the optimal material distribution in the design space is sought through the analysis of the force transmission path, so as to realize the design of high stiffness and a light weight in the support area. Taking the volume as the constraint condition and the minimum strain energy as the design goal, a mathematical optimization model is established, as shown in Formula (3).

(3) $$\begin{array}{l} \min:J\left(\alpha \right)=U^{T}{KU}_{i}\\ s.t.\{\begin{array}{c} KU=F\\ V\left(\alpha \right)=fV_{0}\\ 0\leq \alpha_{i}\leq 1 \end{array} \end{array}$$

where J is the strain energy of the mirror support region, K is the global stiffness matrix, U is the global displacement vector, F is the global load vector, V is the volume constraint of the design domain, f is the volume fraction, and $V_{0}$ is the volume of the design domain.

## 3. Lightweight and High-Stiffness Metal Optical System

### 3.1. Index Requirements

The specific design index requirements of the optical system are shown in Table 1. The spectrum band of the optical system is long-wave infrared (LWIR), and the selected LWIR detector index is shown in Table 2. Since the selected detector is a cooling detector, there is a requirement for the cold stop efficiency, which needs to reach 100%. The weight requirement of the primary mirror and secondary mirror assembly is limited to below 100 g.

### 3.2. Optical System Design

To achieve the compact and lightweight nature of the optical system, the coaxial catadioptric structure was adopted. Considering that the relay lens group composed of the lens was placed in the Dewar of the infrared detector, the number of elements of the optical system were as few as possible, and the number was less than or equal to three. Considering that the first lens of the relay lens group is also the window of the detector Dewar, the aperture and interval of the relay lens group should be as compact as possible. The above series of requirements bring certain challenges to the optical system design.

**Figure 1:**
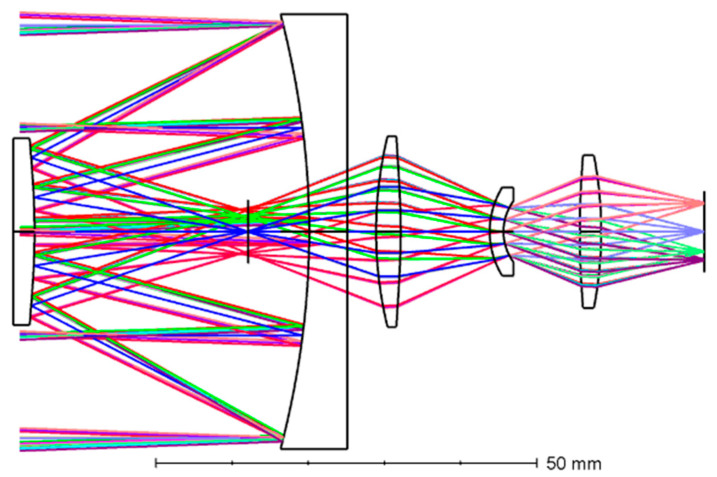
A 2D diagram of the optical system.

**Figure 2:**
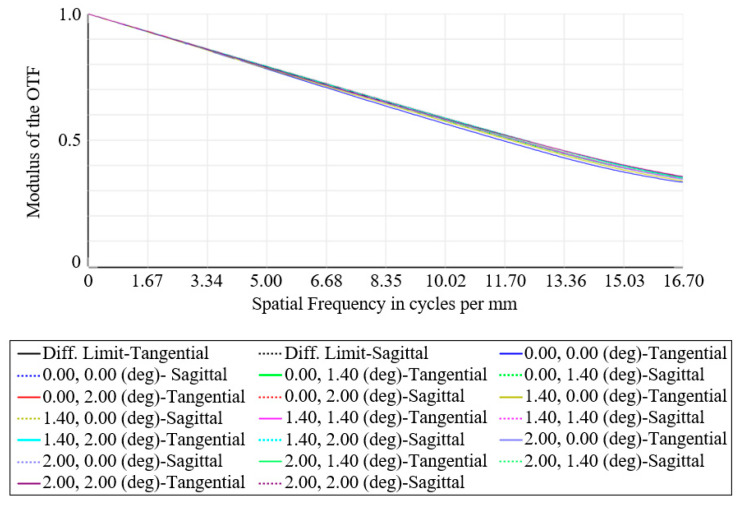
MTF curves of the optical system.

A two-dimensional diagram of the optical system is shown in Figure 1. The optical system is composed of a primary mirror, a secondary mirror, and a relay lens group, in which the primary mirror and the secondary mirror are high-order aspheric surfaces. The relay lens group consists of three lenses, the positive power material is germanium, the negative power material is zinc selenide, and the cold stop is placed on the rear surface of lens 2. To minimize the impact of self-radiation on detection performance, the primary focus of the design is achieving proper stop matching, and the cold stop efficiency should reach 100%. The central obscuration affects the enclosed energy and optical transfer function, and the surface obscuration ratio of the system was 18.3% after design. The MTF curves are shown in Figure 2, from which it can be seen that the imaging quality of each field point in the full field of view reaches the diffraction limit.

### 3.3. Optical Mechanical Structure Design

In the detailed optimization design process, for the mirror in this paper, according to the assumption of thin plate, quantitative analysis and prediction of the root mean square (RMS) of the primary mirror surface were carried out, as shown in formula (4).

(4) $$\delta_{\mathrm{RMS}}=C\frac{\rho g}{E}\frac{r^{4}}{{h_{b}}^{2}}\left(1-v^{2}\right)\#$$

where C is the constant of the mounting conditions, ρ is the material density, g is the gravitational acceleration, r is the semi-diameter of the mirror, v is Poisson’s ratio, and $h_{b}$ is the equivalent bending thickness.

A characterization function of the surface density of the mirror was introduced to achieve a high light-weight ratio of the mirror, as shown by

(5) $$\frac{m}{A}=\rho \left(t_{f}+\eta h_{c}\right)\#$$

where, m/A is the areal mass density, $t_{f}$ is the face sheet thickness, η is the solidity ratio, and $h_{c}$ is the isogrid cell depth.

Limited by the processing technology, traditional mirrors cannot be structurally closed in design, which will inevitably lead to losses in structural rigidity, and it is difficult to achieve a high-rigidity design in the true sense. Here, the lattice filling method of the specific structure was introduced to realize the ultra-lightweight design of the primary mirror; meanwhile, based on the equivalent analysis method, the topology optimization design of the primary mirror assembly was carried out.

**Figure 3:**
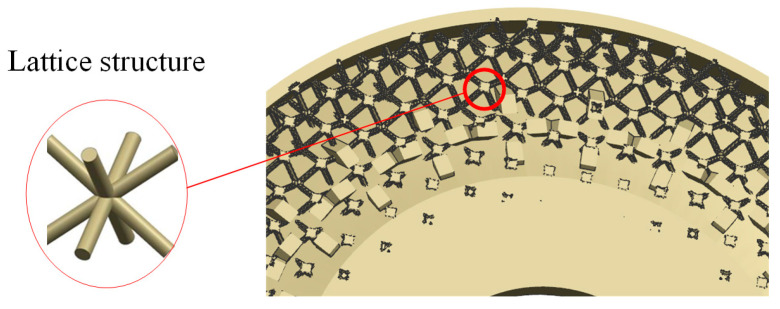
Lattice structure.

As for the lattice, in the lattice structure, under the reasonable setting of the thickness of the mirror surface, we filled the sealing cavity by forming a sealing cavity structure between the mirror surface and the back of the mirror, using a micro-sized lattice to fill the sealing cavity. There are various forms of the lattice structure, such as the periodic structure of the honeycomb unit [11]. In this paper, the lattice was designed concerning the high stability of the space truss structure. This structural form provides uniform support for the mirror surface and ensures high surface stability. The lattice structure is shown in Figure 3.

**Figure 4:**
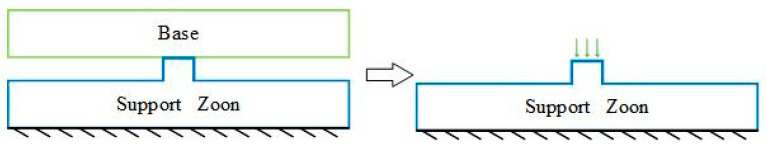
Equivalent analysis and design ideas of the mirror assembly.

The detailed design of the mirror substrate has been completed previously, and the topology optimization design will be carried out in the following, in order to simplify the topology optimization boundaries and to speed up the solution. We adopted the equivalent analysis method. The equivalent analysis method is shown in Figure 4. First, the lattice form of the closed structure on the back of the mirror is given. Meanwhile, the theoretical estimation of the mirror surface accuracy was completed. Then, based on the force transfer path from external loads to the mirror surface, we isolated the mirror assembly and make the mirror base (the mirror surface and the enclosed structure behind the mirror surface) equivalent to a rigid mass point, which was loaded on the mirror support structure through a load form. Setting the external constraint boundary conditions and using the mirror support structure as the design area for topology optimization design, wed achieve a high mechanical performance in the mirror support area while ensuring the stability of surface accuracy.

**Figure 5:**
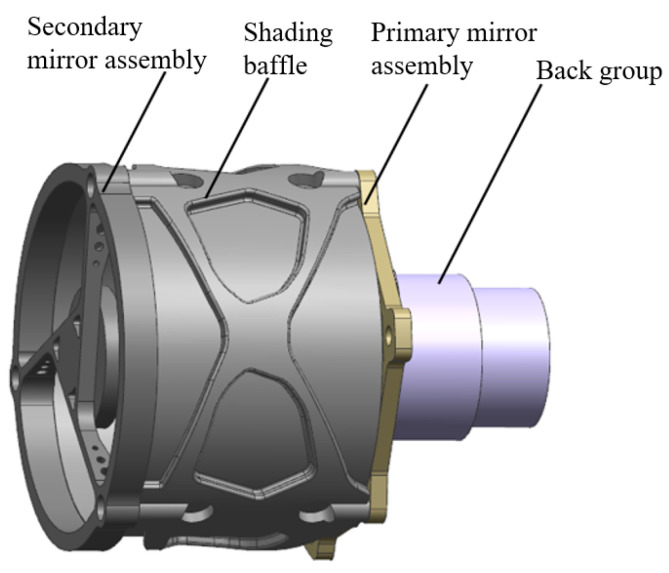
The diagram of the optical-mechanical system.

The structure of the optical system consists of two parts: the front group and the back group. The front group includes primary mirror assembly, shading baffle, and secondary mirror assembly. The back group includes the lens group and lens shell. In order to achieve the ultra-light structure design and considering the interface relationship between the optical system and the overall, the topology optimization method was used to design the front group, and the process was realized by 3D printing. The designed system structure is shown in Figure 5.

#### 3.3.1. Primary Mirror Assembly Design

**Figure 6:**
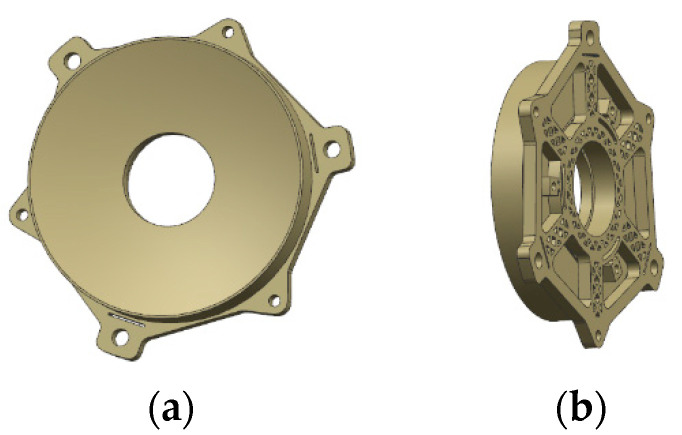
Primary mirror assembly. (**a**) Front view; (**b**) Side view.

**Figure 7:**
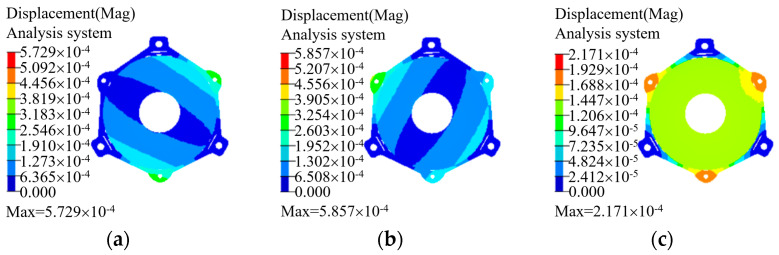
The surface node displacement of the primary mirror under self-gravity. (**a**) Radial direction (X); (**b**) radial direction (Y); (**c**) axis direction (Z).

In order to achieve the minimum number of parts and avoid the external force introduced in the installation process, the primary mirror and its backplane were designed in an integrated way, and a flexible design is added between the mirror body and the support, which effectively plays the role of isolating the stress caused by external force and temperature change. We ran the Optistruct optimization module in Hyperworks software 10.0 to carry out the topology optimization design of the initial structure. In order to ensure the surface shape accuracy of the primary mirror, combined with the lattice structure of the mirror body inside the moderate filling, the mirror stiffness was effectively improved under the condition of satisfying the lightweight design idea. The primary mirror assembly completed by topology optimization is shown in Figure 6, and the surface node displacement under the condition of self-weight is shown in Figure 7 by means of finite element analysis (unit is in millimeters). This emphasizes that the gravity load is applied by means of static loading at the center of mass of the structure, and all the asymmetries in the displacements were due to the fact that the mirror support is in the form of a three-point support, which is not symmetrical in the radial direction. The fitting analysis of the surface shape of the primary mirror shows that the RMS of the surface shape of the primary mirror is 3 nm (about λ/210, λ = 632.8 nm), which has little impact on the imaging quality and can be ignored.

#### 3.3.2. Secondary Mirror Assembly Design

**Figure 8:**
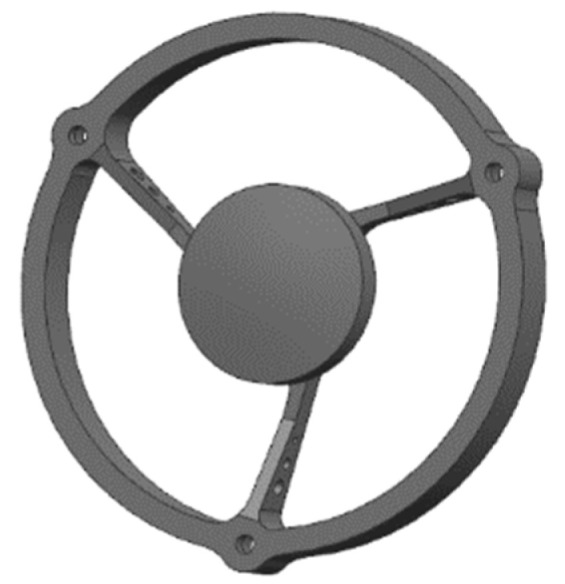
Secondary mirror assembly.

Similar to the form of the primary mirror assembly, the secondary mirror and its support were designed in an integrated way, and the form of the support rids of the secondary mirror assembly was optimized in multiple rounds. The multiple hollow designs inside the assembly and the radial three-rids support scheme were adopted, and the three ribs ring-cutting distribution effectively reduced the bending stress of the assembly and improved the structure’s ability to withstand strong impact and vibration. After optimization, the radial width of each rib was 1.8 mm, which meets the requirements of optical light transmission and has sufficient stiffness. The structural form of the secondary mirror assembly is shown in Figure 8.

#### 3.3.3. Shading Baffle Design

As a transfer structure connecting the primary mirror assembly and the secondary mirror assembly, the shading baffle needs to bear the weight of the secondary mirror assembly and play the role of shielding stray light.

**Figure 9:**
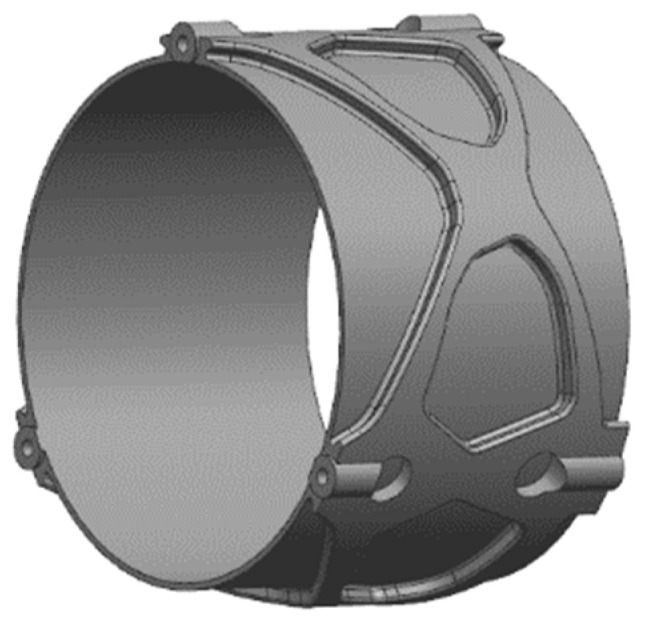
Shading baffle.

In the design, the shading baffle is similar to the primary and secondary components. After topology optimization, the shape and size of the shading baffle are determined. Combined with the requirements of the optical system and considering the processing technology, the wall of the shading baffle was closed. After optimization, the thinnest wall thickness of the shading baffle was 0.8 mm. On the basis of the topology optimization results, the lightweight design was further carried out, and the hollow structure combined with local support was used to further realize the ultra-lightweight design. The structure of the shading baffle is shown in Figure 9.

#### 3.3.4. System Mode and Static Analysis

**Figure 10:**
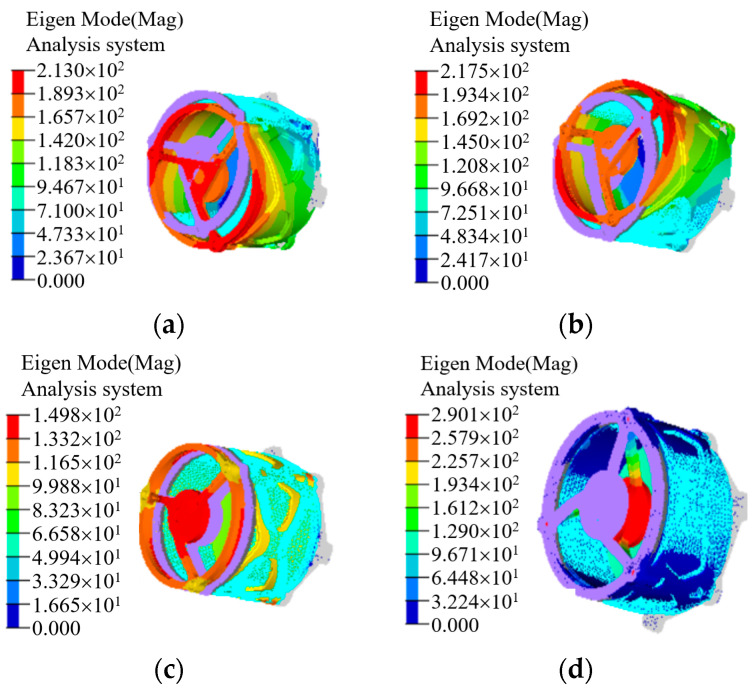
The results of mode analysis. (**a**) First mode (708.3 Hz); (**b**) second mode (708.41 Hz); (**c**) third mode (1213.7 Hz); (**d**) fourth mode (1213.7 Hz).

Mode analysis of the optical system was carried out to verify the stiffness distribution of the whole system and each component. The mode analysis results of the whole machine are shown in Figure 10. The third mode and fourth mode of the system reached 1213.7 Hz, and the stiffness of the whole machine is high.

**Figure 11:**
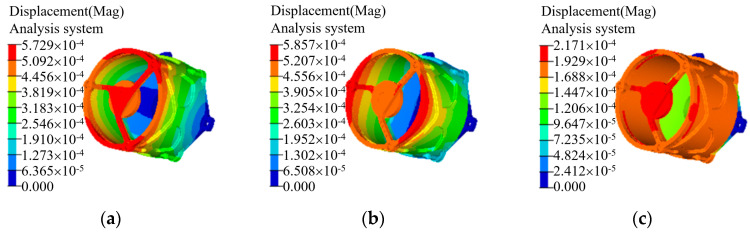
The results of system static analysis. (**a**) X radial direction; (**b**) Y radial direction; (**c**) Z radial direction.

In order to investigate the deformation of the system caused by gravity under its own gravity condition, the static analysis of the system under its own weight was carried out to simulate the actual installation state. The three mounting holes of the back plate of the primary mirror were subjected to six degrees of freedom full constraint treatment, and the deformation of the system under 1 g gravity was simulated. The analysis results are shown in Figure 11. According to the analysis, the maximum synthetic displacement of the system under its own gravity is 5.86 × 10^−4^ mm, which belongs to the sub-micron level. Combined with the mode analysis results, the structure has sufficient stiffness.

#### 3.3.5. Weight Estimation

The weights of the primary mirror assembly, the secondary mirror assembly, the baffle, and the screw were estimated. The total weight was 91.1 g, which meets the requirements of less than 100 g, as shown in Table 3.

## 4. Prototype Development

### 4.1. Additive Manufacturing

**Figure 12:**
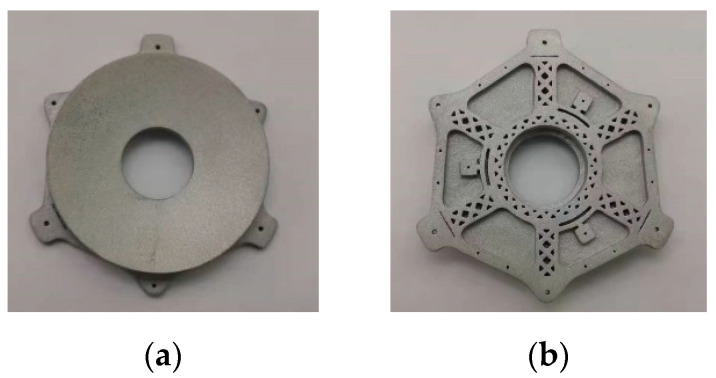
Primary mirror assembly by additive manufacturing. (**a**) Front view; (**b**) side view.

**Figure 13:**
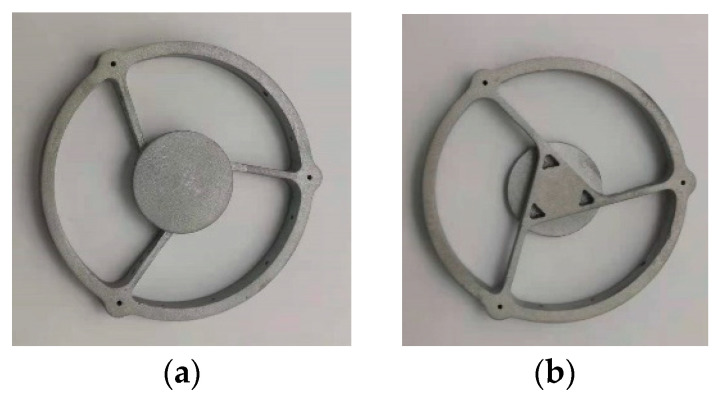
Secondary mirror assembly by additive manufacturing. (**a**) Front view; (**b**) side view.

**Figure 14:**
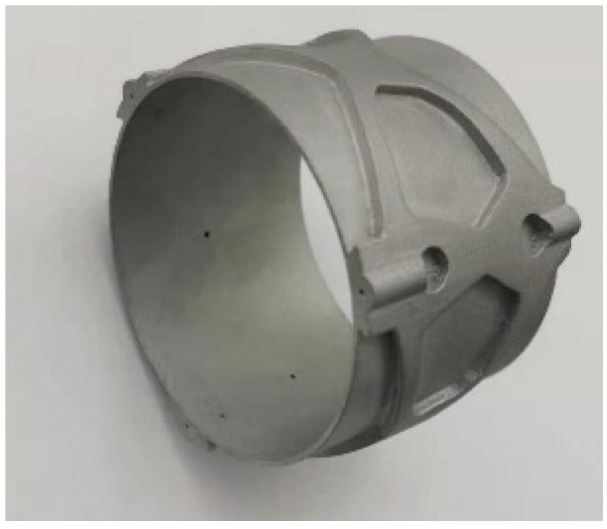
Shading baffle by additive manufacturing.

The primary mirror assembly, secondary mirror assembly, and connecting baffle were 3D printed by additive manufacturing, and system printing was accomplished by a selective laser melting (SLM). Moreover, the residual stress of the mirror assembly was relaxed to ensure the stability of the surface shape after the high-temperature annealing process under vacuum and the mirror material is AlSi_10_Mg. After the printing was completed, the components were subjected to high and low-temperature aging treatment. The printed primary mirror assembly, secondary mirror assembly and shading baffle are shown in Figure 12, Figure 13 and Figure 14, respectively.

### 4.2. Optical Processing

**Figure 15:**
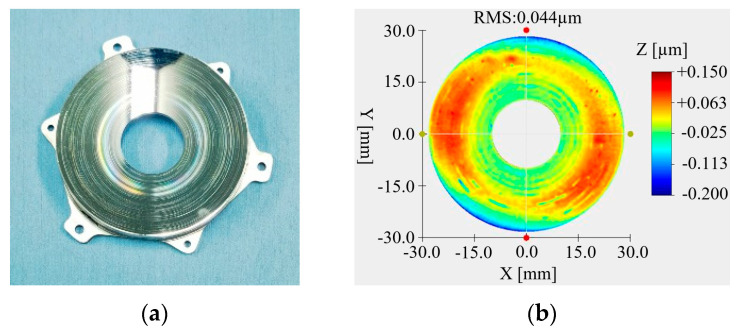
Primary mirror and surface shape data after SPDT. (**a**) Primary mirror; (**b**) surface quality.

**Figure 16:**
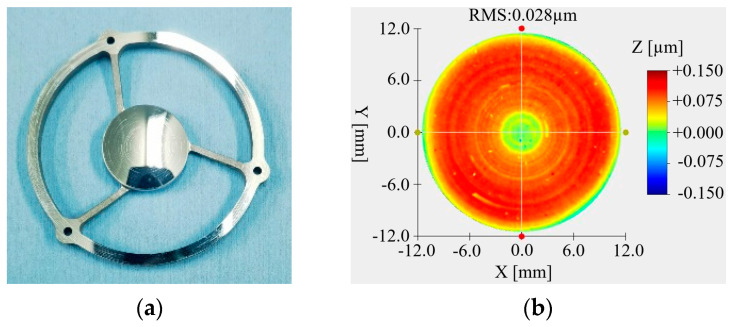
Secondary mirror and surface shape data after SPDT. (**a**) Secondary mirror; (**b**) surface quality.

The primary mirror and secondary mirror were optical processing with SPDT. The primary mirror and secondary mirror after optical processing are, respectively, shown in Figure 15a and Figure 16a. The RMS value of the primary mirror surface reached 0.044 μm, as shown in Figure 15b, and the RMS value of the secondary mirror surface reached 0.028 μm, as shown in Figure 16b. Although the surface shape meets the requirements of use, it can be seen from the diagram that the surface of the mirror has obvious ring bands and dotted spots.

**Figure 17:**
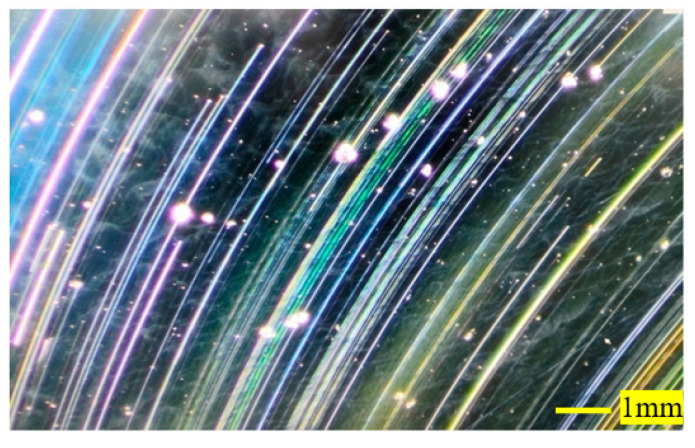
Primary mirror surface under high magnification camera.

Since the surface of the primary mirror and the secondary mirror after optical processing have obvious ring bands and spotted spots, which is significantly different from the surface of the aluminum alloy that is usually not 3D printed, the surface was tested with a high-magnification camera, and the test result is shown in Figure 17. It can be seen from the diagram that the ring bands are very obvious, and the dotted spots are small pits. The size of the largest circular pit in the diagram was measured, and the diameter of the circle reaches 0.3 mm. The above surfaces have a great influence on the enclosed energy of the optical system and can not be used directly. To deal with this problem, the surface of the optical element was modified by nickel–phosphorus alloy plating.

**Figure 18:**
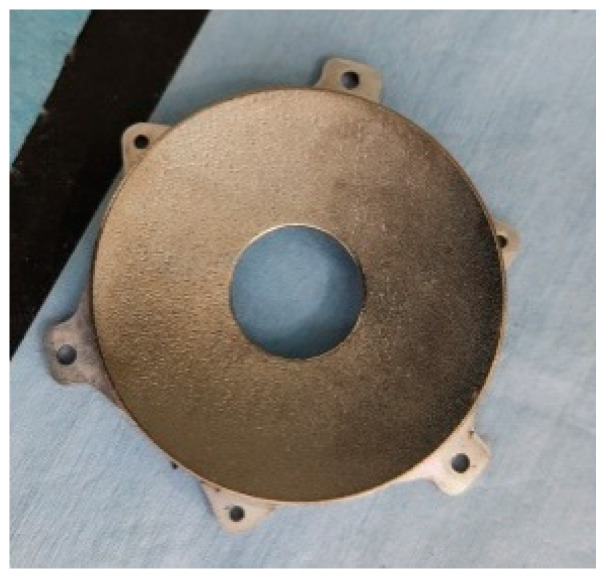
Primary mirror modified by Ni-P.

The optical surfaces of the primary mirror and the secondary mirror after optical processing were modified and a layer of nickel–phosphorus was plated on the surface. In order to prevent the nickel–phosphorus from entering the interior of the 3D-printed metal mirror, the surface of the powder discharge hole of the metal mirror was protected. To make the modified layer dense, the mirror was treated by sandblasting. Figure 18 shows the modified primary mirror with nickel–phosphorus.

**Figure 19:**
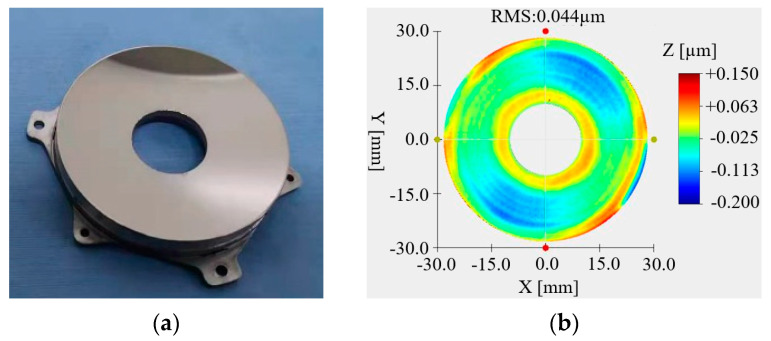
Primary mirror and surface shape data after optical processing. (**a**) Primary mirror; (**b**) surface quality.

**Figure 20:**
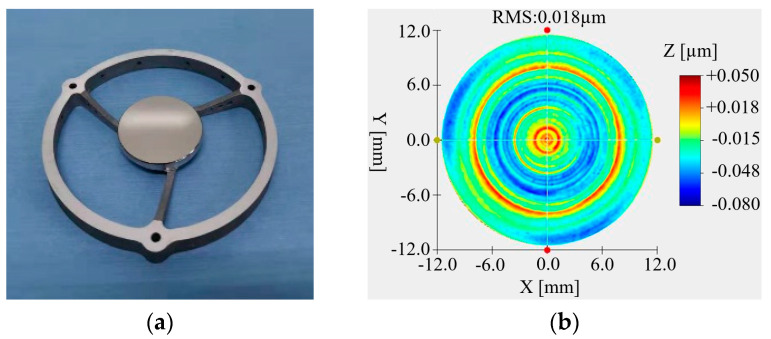
Secondary mirror and surface shape data after optical processing. (**a**) Secondary mirror; (**b**) surface quality.

After the nickel–phosphorus modification was completed, the primary mirror and the secondary mirror were optically processed again. The processed primary mirror and the secondary mirror are shown in Figure 19a and Figure 20a, respectively. It can be seen from the figure that the surface quality of the mirror was significantly improved. The RMS value of the primary mirror is 0.044 μm, as shown in Figure 19b. The RMS value of the secondary mirror is 0.018 μm, as shown in Figure 20b.

**Figure 21:**
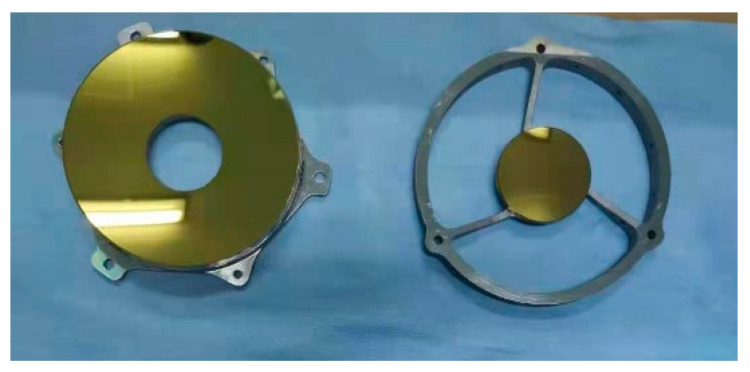
Primary and secondary mirrors after gold-plating.

**Figure 22:**
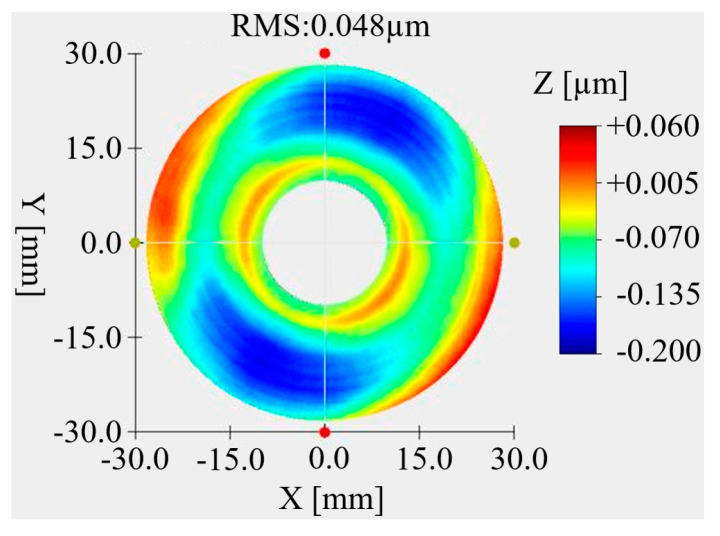
The surface quality of the primary mirror after gold-plating.

**Figure 23:**
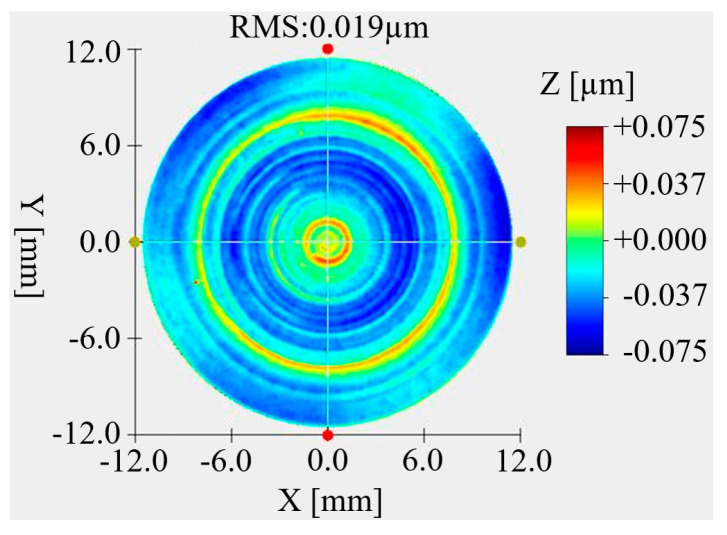
The surface quality of the secondary mirror after gold-plating.

After the optical processing was completed, the primary mirror and the secondary mirror were coated with gold film. The primary and secondary mirrors after coating are shown in Figure 21. The surface shape test on the primary mirror and the secondary mirror after coating was carried out. The surface shape test results of the primary mirror are shown in Figure 22. The RMS value of the primary mirror surface shape is 48 nm. Compared with that before coating, it changed by 4 nm. Considering the measurement error, there was no change in the surface shape before and after coating. The surface shape test results of the secondary mirror are shown in Figure 23. The RMS value of the secondary mirror surface shape is 19 nm. Compared with the 18 nm before coating, the change is 1 nm. Considering the measurement error, the surface shape showed no change before and after coating.

### 4.3. Optical Mechanical Assembly

**Figure 24:**
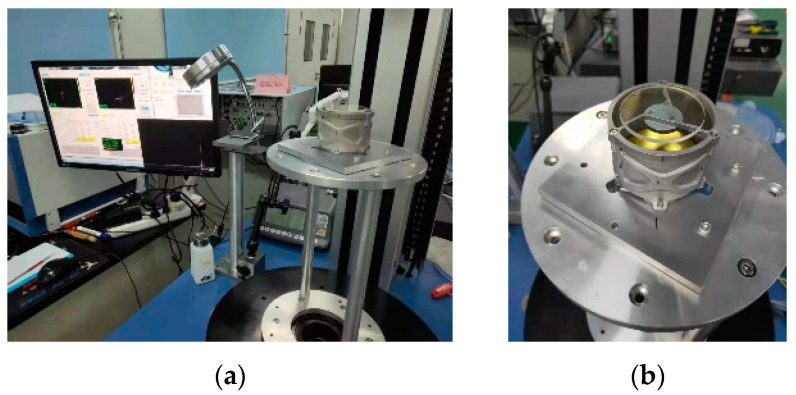
Centering alignment of primary and secondary mirror assembly. (**a**) Alignment worksite; (**b**) mirrors after centering.

Using the centering instrument, the primary mirror assembly and the secondary mirror assembly were centering aligned, as shown in Figure 24.

### 4.4. Performance Testing

**Figure 25:**
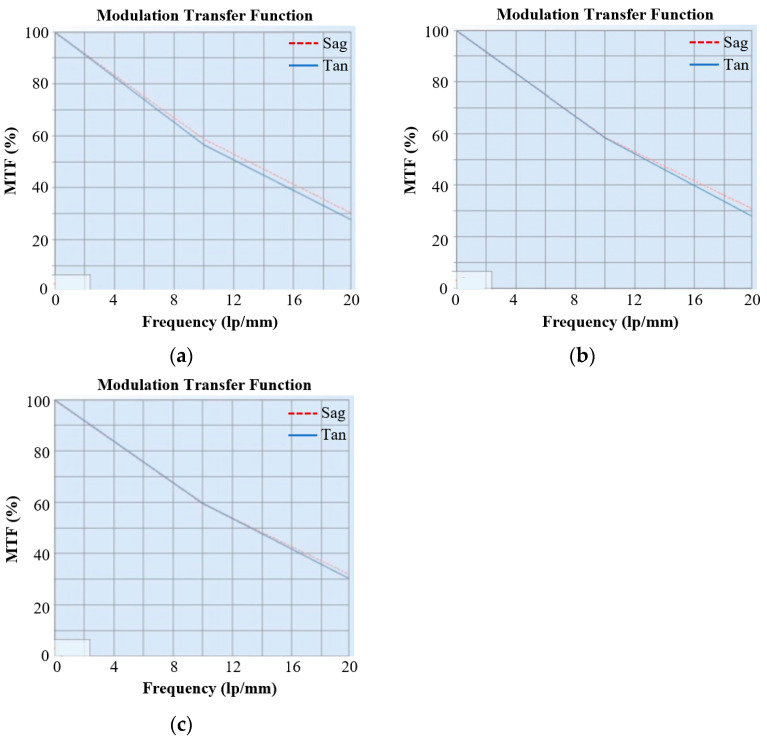
MTF test results for different FOVs. (**a**) The MTF curves of central FOV; (**b**) the MTF curves of 0.7 FOV; (**c**) the MTF curves of 1 FOV.

After the assembly was completed, the core indicators were tested by the transfer function tester. The focal length test result of the optical system was 108.26 mm, and the deviation from the design value of 110 mm was 1.74 mm, within the tolerance of ±3%. The MTF of the optical system at different fields of view was tested. The MTF curves of the central field of view, 0.7 field of view, and 1 field of view are given in Figure 25. The test values at the Nyquist frequency of 16.7 lp/mm are all greater than 0.35, which is close to the diffraction limit.

The weights of the primary and secondary mirror assembly were measured, with a weight of 96.04 g. Detailed weight data of each component are shown in Table 4, and were 4.94 g higher than the estimated weight of 91.1 g in Table 3. The primary mirror assembly increased by 4.04 g, and the weight increase was mainly caused by 3D-printing deviation, nickel–phosphorus modification, and gold-plating. The weight of the shading baffle increased by 0.87 g, and the actual size of the 3D printing was slightly different from the theoretical design. The quality of secondary mirror assembly and screws was basically the same. The weight of the component met the technical requirement of less than 100 g.

## 5. Conclusions

In this paper, a local cooling catadioptric optical system was specifically designed for the detection of low-temperature targets in deep space. The lens group was situated within the Dewar of the infrared detector. The optimization process employed the topology optimization method to refine the primary and secondary mirror assembly. Additionally, additive manufacturing techniques were used to fabricate the primary mirror assembly, secondary mirror assembly, and connecting baffle. The optical components were then processed using single-point diamond turning. To address surface defects from the printing process, the nickel–phosphorus modification method was applied, followed by single-point processing to enhance the mirror’s surface quality. The test results indicated the high accuracy of the mirror’s surface shape. The focal length of the optical system was 108.26 mm, which meets the requirements of the index. The modulation transfer function in the full field of view reached the diffraction limit, and the system’s weight was impressively low at just 96.04 g, well below the specified limit of less than 100 g. Through the development of the whole machine, it is fully demonstrated that the metal additive manufacturing method can be used as an effective means to significantly enhance the performance of optical systems.

## Figures and Tables

**Table 1:** Design index of the optical system.

Parameters	Index Requirements
Waveband	8–10 μm
F/#	2
Focal length	110 mm
Field of view	4° × 4°
Cold stop efficiency	100%
Weight of primary and secondary mirror assembly	Less than 100 g

**Table 2:** Index of LWIR detector.

Parameters	Index Data
Resolution of resolution	256 × 256
Pixel size	30 μm × 30 μm
F/#	2

**Table 3:** Weight estimation.

Name	Weight (g)
Primary mirror assembly	40.6
Secondary mirror assembly	16.8
Shading baffle	32.2
Screws	1.50
Total	91.1

**Table 4:** Weight test.

Name	Actual Weight (g)
Primary mirror assembly	44.64
Secondary mirror assembly	16.8
Shading baffle	33.07
Screws	1.53
Total	96.04

## Data Availability

The data presented in this study are available on request from the corresponding author.
